# Cardiac Overexpression of XIN Prevents Dilated Cardiomyopathy Caused by *TNNT2* ΔK210 Mutation

**DOI:** 10.3389/fcell.2021.691749

**Published:** 2021-06-17

**Authors:** Bin Li, Yifan Guo, Yongkun Zhan, Xinyan Zhou, Yongbo Li, Chao Zhao, Ning Sun, Chen Xu, Qianqian Liang

**Affiliations:** ^1^Department of Physiology and Pathophysiology, School of Basic Medical Sciences, Fudan University, Shanghai, China; ^2^Shanghai Institute of Precision Medicine, Ninth People’s Hospital, Shanghai Jiao Tong University School of Medicine, Shanghai, China; ^3^Department of Medical Microbiology and Parasitology, School of Basic Medical Sciences, Fudan University, Shanghai, China; ^4^Shanghai Key Laboratory of Clinical Geriatric Medicine, Institute of Integrative Medicine, Fudan University, Shanghai, China

**Keywords:** dilated cardiomyopathy, gene therapy, *TNNT2*, cardiac remodeling, XIN

## Abstract

*TNNT2* mutation is associated with a range of cardiac diseases, including dilated cardiomyopathy (DCM). However, the mechanisms underlying the development of DCM and heart failure remain incompletely understood. In the present study, we found the expression of cardiac XIN protein was reduced in *TNNT2*-ΔK210 hESCs-derived cardiomyocytes and mouse heart tissues. We further investigated whether XIN protects against *TNNT2* mutation-induced DCM. Overexpression of the repeat-containing isoform XINB decreased the percentage of myofilaments disorganization and increased cell contractility of *TNNT2*-ΔK210 cardiomyocytes. Moreover, overexpression of XINB by heart-specific delivery via AAV9 ameliorates DCM remodeling caused by *TNNT2*-ΔK210 mutation in mice, revealed by partially reversed cardiac dilation, systolic dysfunction and heart fibrosis. These results suggest that deficiency of XIN may play a critical role in the development of DCM. Consequently, our findings may provide a new mechanistic insight and represent a therapeutic target for the treatment of idiopathic DCM.

## Introduction

Heart failure due to cardiomyopathy is among the most common causes of cardiovascular mortality (Harmon et al., 2005). Numbers of mutations in genes encoding sarcomeric proteins have been identified in individuals with cardiomyopathy (Morita et al., 2005; Villard et al., 2011). Cardiac troponin T (cTnT), encoded by *TNNT2*, is a cardiac muscle specific sarcomere protein that is important for the cell morphology, sarcomere assembly and myofilament contraction in cardiomyocytes (Solaro and Rarick, 1998; Wei and Jin, 2011). Deletion of lysine 210 (ΔK210) in *TNNT2* gene has been found to cause familial dilated cardiomyopathy (DCM) (Kamisago et al., 2000), which is characterized by dilated ventricular chamber and reduced systolic function leading to progressive heart failure with high mortality (Du et al., 2007; Kimura, 2010; Sfichi-Duke et al., 2010). Attempts to elucidate the molecular mechanisms underlying DCM mutations showed multiple molecular mechanisms including Ca^2+^ sensitivity of myofilament (Morimoto et al., 2002; Sfichi-Duke et al., 2010), remodeled intracellular calcium handling (Inoue et al., 2013; Lan et al., 2013) or interactions among the thin filaments protein constituents (Morimoto et al., 2002; Lombardi et al., 2008; Kimura, 2010) were involved in development of the disease. However, the detailed pathogenic mechanism underlying DCM disease remains poorly understood. Combined with human pluripotent stem cell (hPSC) technology and efficient cardiac differentiation protocols, human cardiomyocytes with corresponding gene mutations could be obtain *in vitro*, making it possible to expediently explore the effects of genetic mutations in the initial phases of disease development using isogenic human disease model systems (Sallam et al., 2014; Savla et al., 2014).

Xin actin-binding repeat-containing proteins (XIRPs) are a family of striated muscle-specific proteins defined by the presence of 15–28 copies of the conserved 16-aa XIN repeats. XIN is encoded by XIRP1 gene, also called *hXin*α or *CMYA1*, is a striated muscle-specific gene and belongs to the XIRP family (Eulitz et al., 2013). Human XIRP1 gene gives rise to three XIN isoforms, known as XINA, XINB, and XINC (Molt et al., 2014). It is interesting that this gene has only been found in vertebrates with true chambered heart. XIN is expressed during early developmental stages of cardiac and skeletal muscles (Pacholsky et al., 2004). In cardiomyocytes, XIN is localized predominantly at intercalated discs (ICDs), which showed significance in the regulation of cardiac development and functions (Sinn et al., 2002). Multiple binding sites enabled XIN strengthen membrane apposition between cardiomyocytes by linking actin cytoskeleton to adherens junctions of ICDs (van der Ven et al., 2006; Wang et al., 2012, 2013). Additionally, its localization at adherens junctions of muscle cells and interaction with β-catenin, filaminC, and Mena/VASP imply a role in linking and transducing signals important for cardiac remodeling. Inactivation of *Xin* in chick embryos led to looping defects, abnormal beating behavior and edema (Wang et al., 1999), while *Xin*-deficient mice display myopathy, impaired contractility, and attenuated muscle repair (Al-Sajee et al., 2015), which indicating a crucial role in heart development and cardiac disease. Moreover, it has been observed that Xin may participate in a BMP-Nkx2.5-MEF2C pathway and promote the expression of ventricular myosin heavy chain (vMHC) gene to participate in cardiogenesis regulation (Wang et al., 1999, 2014). All these indicating an important role for XIN in early myofibrillogenesis and later in the maintenance of striated muscle integrity.

In the previous study, we characterized that the cellular phenotypes of *TNNT2*-ΔK210 hESC-CMs (human embroynic stem cells dervived cardiomyocytes) recapitulated DCM disease features (Li et al., 2021). Through whole transcriptomic analysis, down-regulated expression of XIN was identified in day35 *TNNT2*-ΔK210 hESC-CMs. In this study, we further confirmed the decreased expression of XIN in DCM models and focused on the therapeutic effects of XIN. We found that overexpression of XINB improved sarcomere organization and contraction force of *TNNT2*-ΔK210 cardiomyocytes. *In vivo* cardiac-specific overexpression of XINB via AAV9 in neonatal *TNNT2*-ΔK210 DCM mice significantly rescued the DCM phenotypes. In conclusion, our findings uncovered the importance of XIN in *TNNT2* mutation induced dilated cardiomyopathy.

## Materials and Methods

### Cell Culture and Cardiac Differentiation

The human ESC line H7 obtained from WiCell Research Institute under specific Material Transfer Agreement was used in this study. All human stem cells researches followed the ISSCR Guidelines for the Conduct of Human Embryonic Stem Cell Research. Human ESCs were cultured using mTesR human pluripotent stem cell culture medium (STEMCELL Technologies) on Matrigel-coated (BD Biosciences) 6-well or 12-well plates. Cardiac differentiation were performed according to previously described protocols (Lian et al., 2013). Each of three congenic lines were used in the following analysis. hESC lines between 70 and 80 passages were used in the following analysis.

### RNA Extraction and Real Time Quantitative PCR

Total RNA was extracted using Trizol from Invitrogen according to the operating manual. The relative expression profiles of *XIRP1* were examined by TOYOBO real-time PCR chemistry. Glyceraldehyde-3-phosphate dehydrogenase (*GAPDH*) was used as endogenous control gene. PCR reaction were run in a final volume of 20 μl composed of 1 μl of cDNA, 9 μl of buffer Mix and 100 nM primer pairs. The PCR condition consisted of one initial denaturation at 95°C for 4 min, followed by 40 cycles of denaturation at 95°C for 15 s and annealing at 60°C for 1 min, which were performed by Bio-Rad iQ5 Real Time PCR System. All reactions were performed in triplicate. The primer sequences used are provided in Supplementary Table 1.

### Western Blot

Cells or heart tissue were lysed with RIPA buffer (50 mM Tris pH 7.4, 150 mM NaCl, 1% Triton X-100, 1% sodium deoxycholate, 0.1% SDS) (Beyotime Biotechnology, P0013B) with protease inhibitor cocktail (APExBIO, K1012). Protein concentrations were determined by BCA Protein Assay Kit (TIANGEN, PA115). Equal amounts of protein were loaded on 10% SDS-PAGE. After electrophoresis, proteins were transferred to PVDF membrane (Millipore, IPVH00010). After blocked with 5% skimmed milk at room temperature, membranes were incubated with primary antibodies (Xirp1: sc-166658, Santa Cruz Biotechnology, diluted at 1:1,000; GAPDH: 60,004-1-Ig, Proteintech, diluted at 1:1,000; β-tubulin: 10068-1-AP, Proteintech, diluted at 1:2,000) at 4°C overnight and then incubated with corresponding secondary antibody. Western blots results were detected with Tanon 5200 Multi.

### Immunofluorescence Staining

Morphological changes of hESC-cardiomyocytes were detected using immunostaining. After fixed with 4% paraformaldehyde and permeabilized with 0.05% Triton X-100, the cells were blocked with 4% goat serum, incubated with primary antibodies (cTnT: ab45932, Abcam, diluted at 1:400; Xirp1: sc-166658, Santa Cruz Biotechnology, diluted at 1:100) overnight at 4°C, and then visualized with secondary antibody. Frozen left ventricular tissue slides were incubated with Alexa Fluor^TM^ 488 Conjugated-Wheat Germ Agglutinin (Life Technologies, United States) and DAPI (Life Technologies) to visualize myocyte membranes. Fluorescence images were obtained with a confocal microscope (LEICA SP8).

### Echocardiography

Echocardiography was performed at determined time. Mice were anesthetized by 1.5% isoflurane and M-mode images were captured and measured using the small animal echocardiography analysis system at the midventricular level. The echocardiographer was blinded to mouse genotypes or treatments.

### Histological Analysis

Cardiac tissue from mice at 3 months of age was fixed in 4% formaldehyde and paraffin embedded. The sections were sliced at 5 μm. Hematoxylin-eosin (H&E) and Masson staining were performed to examine general histology and analyze cardiac fibrosis, respectively. The sections were observed and photographed by the microscope. For the detection of transmission electronmicroscopy, heart tissues from ventricle were immediately fixed in 2.5% glutaraldehyde solution for about 1 h at room temperature and post-fixed for 3 h. Samples were embedded, sectioned at about 70 nm thickness, and stained with lead citrate for 8 min. Micrographs were captured using a PHILIPS CM-120 transmission electron microscope.

### Lentivirus Production

XINB cDNA was cloned into target gene lentiviral vector (pCDH-EF1-MCS-T2A-copGFP) at the MCS locus. The HEK293T cells were transfected with the target gene lentiviral vector plasmid and packaging plasmids using Lipofectamine 3,000 (Life Technologies). The transfected cells were cultivated and collected supernatant every 24 h. Ultrafiltration was used to concentrate the medium. Lentiviruses expressing EGFP gene were used as the control. The efficiency of virus transduction was confirmed 24 h after infection.

### Recombinant AAV9 Virus Production

We cloned XINB or Luciferase cDNA into ITR-containing AAV9 plasmid with cardiac specific TNNT2 promoter and obtained the pAAV:TNNT2:XINB and pAAV:TNNT2:Luciferase plasmids. AAV9 was packaged using HEK293 cells with AAV9:Rep-Cap and pHelper and purified by an iodixanol gradient centrifugation. AAV9 titer was determined by quantitative PCR. 50 μl containing 1 × 10^12^ vg AAV9-Luci or AAV9-XINB virus were used for one mouse. Intraperitoneal injection of AAV9 was performed within 3 days of birth.

### Contraction Force Measurements

HESC-cardiomyocytes were separated into single cell and cultured on a 0.4–0.8 mm thick mattress of undiluted Matrigel for 2–3 days. We used video-based edge detection to measure contractility, which were visualized using a Zeiss CFM-500 inversion fluorescence microscope. Spontaneous contraction traces were recorded and analyzed by FelixGX software (PTI).

### *In vivo* Bioluminescence Imaging

*In vivo* bioluminescence imaging was performed to confirm AAV-9 mediated gene expression. 15 min after intraperitoneal injection of D-luciferin (Promega, 15 mg/ml, 0.2 ml) under anesthesia with inhalation of isoflurane, the mice were placed for imaging using an IVIS Lumina K system (PerkinElmer).

### Animals

A knock-in mouse with deletion of Lys-210 mutation in cardiac troponin T gene (*TNNT2*-ΔK210) was used as the DCM model animal, which were constructed by VIEWSOLID biotechnology co., LTD. All procedures involving animal use, housing, and operations in this study were approved by the Institutional Animal Care and Use Committee of the Fudan University and met the Guide for the Care and Use of Laboratory Animals (National Institutes of Health).

### Statistical Analysis

All data are presented as mean ± SEM (standard error of the mean). Two normally distributed data sets analysis was performed using unpaired two-tailed Student’s *t*-test. One-way ANOVA was used to test for significant differences in multiple group comparisons. *P* < 0.05 was considered statistically significant. *^∗^P* < 0.05, *^∗∗^P* < 0.01, ^∗∗∗^*P* < 0.001.

## Results

### XIN Deficiency Was Detected in the TNNT2-ΔK210 hESC-Derived Cardiomyocytes

**FIGURE 1:**
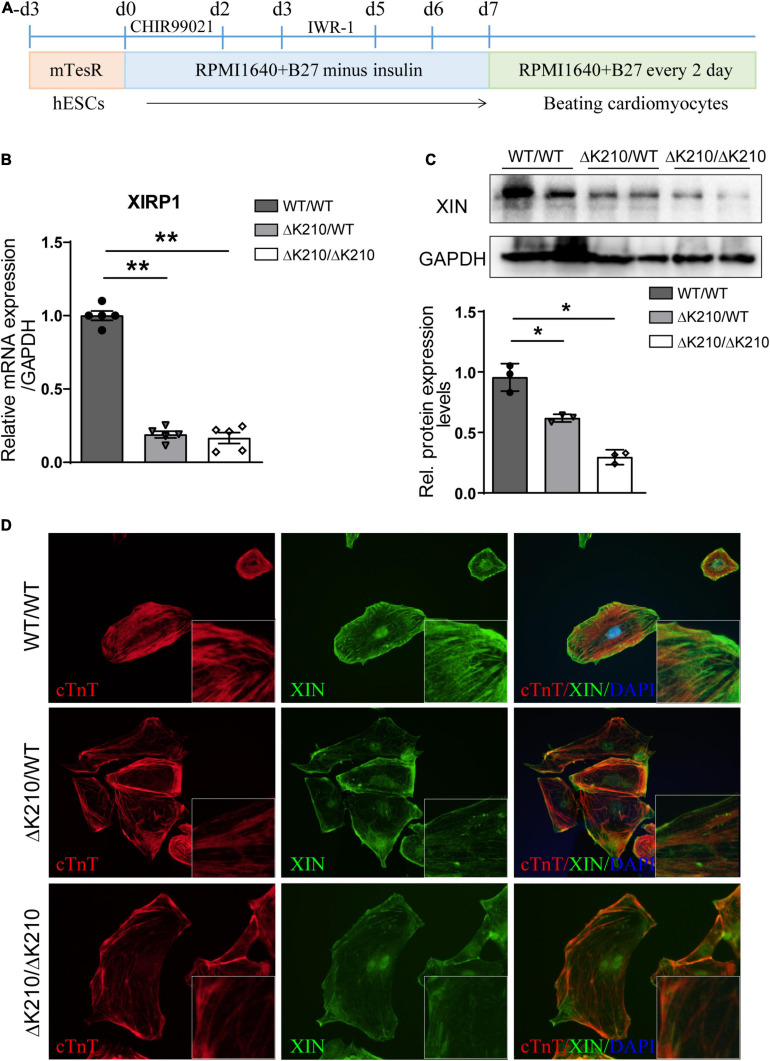
Cardiac differentiation of *TNNT2*-ΔK210 mutant hESCs and the expression of XIN. **(A)** Schematics of optimized chemically defined cardiac differentiation protocol. **(B)** Quantitative PCR verification of *XIRP1* expression in wild type (WT/WT), *TNNT2*-ΔK210 (heter: ΔK210/WT, homo: ΔK210/ΔK210) hESC-cardiomyocytes at day35 post cardiac differentiation. **(C)** Detection of XIN protein expression at day35 post differentiation by Western blotting. GAPDH was used as the loading control. **(D)**, Immunostaining detection of XIN (green) and cTnT (red) in cultured WT/WT, WT/ΔK210 and ΔK210/ΔK210 cardiomyocytes. Data are represented as mean ± SEM. *P < 0.05, **P < 0.01.

HESCs (H7) lines with either a DCM-causing heterozygous ΔK210 (ΔK210/WT) or homozygous ΔK210 (ΔK210/ΔK210) mutation in the *TNNT2* gene were generated as described before (Li et al., 2021). Through modification of the WNT signaling pathway, we differentiated the isogenic heterozygous and homozygous *TNNT2*-ΔK210 mutant hESCs into cardiomyocytes and beating cells were observed on day 8∼9 after cardiac differentiation (Figure 1A). Immunostaining of WT, and *TNNT2*-ΔK210 hESCs-derived cardiomyocytes showed positive staining of cardiac markers cTnT, α-actinin and MLC2V (Supplementary Figure 1A). Fluorescent activated cell sorting (FACS) analyses for cTnT showed a ∼90% purity of cTnT + cardiomyocytes in our cardiac differentiation and similar efficiencies among H7 WT and *TNNT2*-ΔK210 hESC lines (Supplementary Figure 1B). In the previous study, we characterized that the cellular phenotypes, such as Ca^2+^ handling, and contractility of the *TNNT2*-ΔK210 cardiomyocytes recapitulated DCM disease features (Li et al., 2021). We further analyzed whole transcriptomic results of day35 WT (WT/WT) and *TNNT2*-ΔK210 cardiomyocytes (Supplementary Figure 2A). Since there are no reports of patients carrying homozygous *TNNT2*-ΔK210 mutation, heterozygous *TNNT2*-ΔK210 cardiomyocytes may have a stronger disease-related significance. Through comparing with WT cardiomyocytes and RT-qPCR analysis, we found *XIRP1* was sharply down-regulated and the encoded XIN also decreased in both heterozygous and homozygous *TNNT2*-ΔK210 cardiomyocytes at day35 post cardiac differentiation (Figures 1B,C and Supplementary Figures 2B,C). Previous studies showed XIN is an F-actin-binding protein and expressed in the early stages of development of cardiac muscles, so we hypothesized that reduction of XIN participated in the development of dilated cardiomyopathy. Localization and myofilament pattern of XIN in WT and *TNNT2*-ΔK210 cardiomyocytes were then further examined. Compared to WT cardiomyocytes, staining patterns of XIN combined with myofilaments cTnT were changed and weaker in *TNNT2*-ΔK210 cardiomyocytes (Figure 1D). These results suggested a possible role of XIN in the regulation of cardiomyocytes remodeling.

### XINB Improved Myofilaments Organization in TNNT2-ΔK210 Cardiomyocytes

**FIGURE 2:**
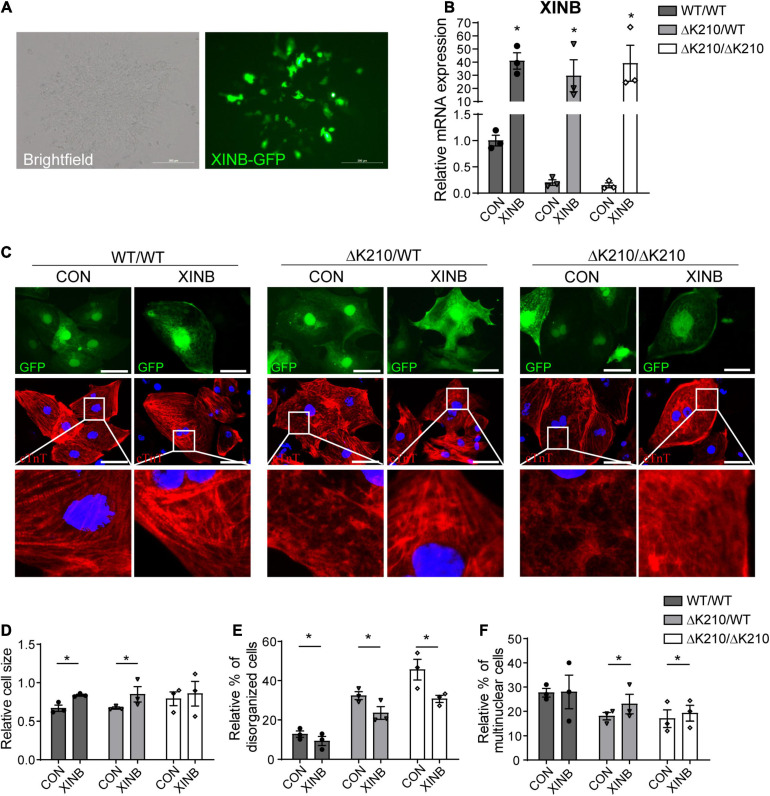
Overexpression of XINB partially rescued the DCM phenotype of *TNNT2*-ΔK210 cardiomyocytes. **(A)** Brightfield and fluorescence images of hESC-cardiomyocytes illustrating green fluorescence protein (GFP) expression after XINB-lentivirus transfection. Scale bars, 200 mm. **(B)** Relative XINB expression in WT/WT, WT/ΔK210, and ΔK210/ΔK210 cardiomyocytes determined by quantitative PCR. **(C)** Representative immunostaining of cTnT (red) in cultured WT/WT, WT/ΔK210, and ΔK210/ΔK210 cardiomyocytes after XINB overexpression. Cells transfected with lentivirus expressing GFP were used as control. Scale bars, 50 mm. **(D)** Quantification of relative cell size and **(E)** statistics of the relative disorganized sarcomeric pattern for WT/WT, WT/ΔK210, and ΔK210/ΔK210 cardiomyocytes after overexpression of XINB. **(F)** Percent of multinuclear cells increased after XINB overexpression in *TNNT2*-ΔK210 cardiomyocytes. *n* > 30, 3 lines in each group; data are represented as mean ± SEM. *P < 0.05.

Because of the limitations of lentivirus and recombinant adeno-associated virus vector capacity, we used isoform of XINB (containing XIN-repeats, peptide motifs which bind actin filaments by coiling around them) in the next *in vitro* and *in vivo* study. We overexpressed XINB in *TNNT2*-ΔK210 cardiomyocytes to check whether restore of XINB can rescue the DCM-like cellular phenotypes. Lentiviral vectors containing XINB-T2A-GFP were generated, with GFP cassette to indicate the transduced cells, while GFP only was used as the negative control. Cardiomyocytes were infected with XINB or control lentiviruses for 7 days. Obvious GFP (Figure 2A) and increased XINB (Figure 2B) were detected after transfection. As XIN binding to actin filaments and may play an important role in early myofibrillogenesis, we then used immunofluorescence to detect the myofilament structure after XINB overexpression. Compared to WT cardiomyocytes, myofibrils were poorly organized with diffusions and damages (myofilament staining was weaker and less dense, punctate distribution of sarcomeric cTnT over one fourth of the total cellular area) (Sun et al., 2012) in ΔK210 cardiomyocytes. While XINB overexpressed ΔK210 cardiomyocytes showed better organized and dense myofilaments over the entire cellular area (Figure 2C). An increase in cell size of WT/WT and ΔK210/WT cardiomyocytes and a reduced percentage of *TNNT2*-ΔK210 cardiomyocytes with disorganized cTnT distribution patterns were observed after XINB expression (Figures 2D,E). We also found higher frequency of multinucleation in XINB overexpressed *TNNT2*-ΔK210 cardiomyocytes versus control (Figure 2F), which might suggest a further mature state. In conclusion, phenotypes of *TNNT2*-ΔK210 cardiomyocytes such as dispersion and disorganization of myofilaments were improved after XINB overexpression. These results showed that XIN may play an important role in myofibrillar assembly and the maintenance of muscle integrity.

### Overexpression of XINB Increased Contractile Force of hESC-Derived Cardiomyocytes

**FIGURE 3:**
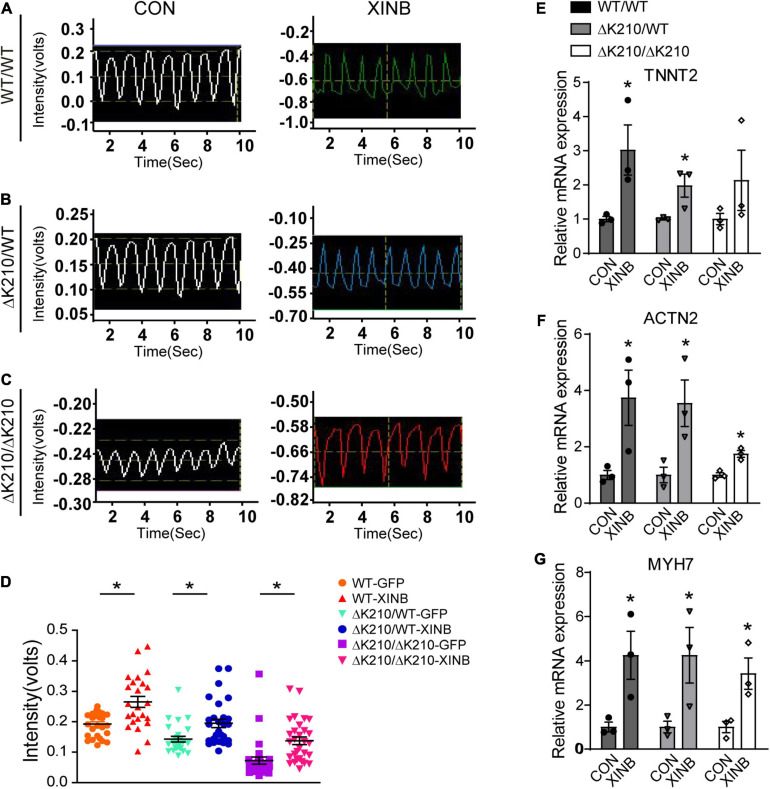
XINB increased contractility of *TNNT2*-ΔK210 cardiomyocytes. **(A)** Representative video recording traces of spontaneous contraction of single WT/WT. **(B)** ΔK210/WT and **(C)** ΔK210/ΔK210 cardiomyocytes in control or XINB overexpression group. **(D)** Statistics of video recording intensities showed increased contractile forces after XINB overexpression in WT/WT (*n* = 29), ΔK210/WT (*n* = 30), and ΔK210/ΔK210 (*n* = 30) cardiomyocytes from 3 independent lines in each group. Relative expression of cardiac muscle contraction related genes **(E)** *TNNT2*, **(F)** *ACTN2*, and **(G)** *MYH7* increased after XINB overexpression. Data are represented as mean ± SEM. *P < 0.05.

To further evaluate the effects of XINB overexpression, we next examined the contractile force of cardiomyocytes. Compared with control, XINB treated WT/WT, ΔK210/WT and ΔK210/ΔK210 cardiomyocytes showed increased contractility (Figures 3A–D). It was previously reported that, in chicken, cXin participated in a BMP2–Nkx2.5–Mef2C–cXin–vMHC pathway to regulate cardiogenesis (Wang et al., 2014). Then we further examined expression of genes such as *TNNT2*, *ACTN2* and *MYH7*, which related to cardiac muscle contraction, and found they were also up-regulated after XINB overexpression (Figures 3E–G), suggesting an improvement in cardiomyocyte contractile function. And the increased expression of cardiac structural genes after XINB transfection may also contribute to the enlarged cell size, improved myofilaments organization and maturation of cardiomyocytes in Figures 2D–F.

### XIN Was Down-Regulated in the TNNT2-ΔK210 Mice Hearts

**FIGURE 4:**
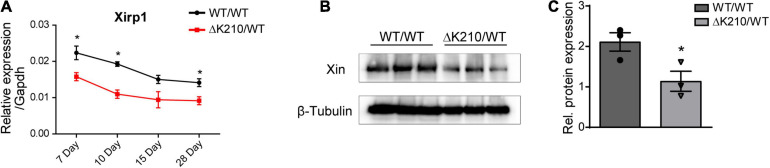
Expression of *Xirp1* in the *TNNT2*-ΔK210 mice hearts. **(A)** Changes in temporal mouse *Xirp1* expression in wild type and *TNNT2*-ΔK210 mutant mice. **(B)** Western blot analysis showed decreased level of XINprotein in Day 7 *TNNT2*-ΔK210 mice hearts. **(C)**, Quantification of western blot signals in **(B)** and normalized to the loading control (β-Tubulin). Data are represented as mean ± SEM. *P < 0.05.

We next detected the expression of Xin in heart tissues of wild type and *TNNT2*-ΔK210 mice at different days after birth. The results indicated that *Xirp1* expression was also down-regulated in the *TNNT2*-ΔK210 mice since they were born (Figure 4A). Western blot analysis showed decreased level of XIN (encoded by *Xirp1*) protein in *TNNT2*-ΔK210 mice hearts 7 days after birth (Figures 4B,C).

### XINB Protected Against Dilated Cardiomyopathy in TNNT2-ΔK210 Mice

**FIGURE 5:**
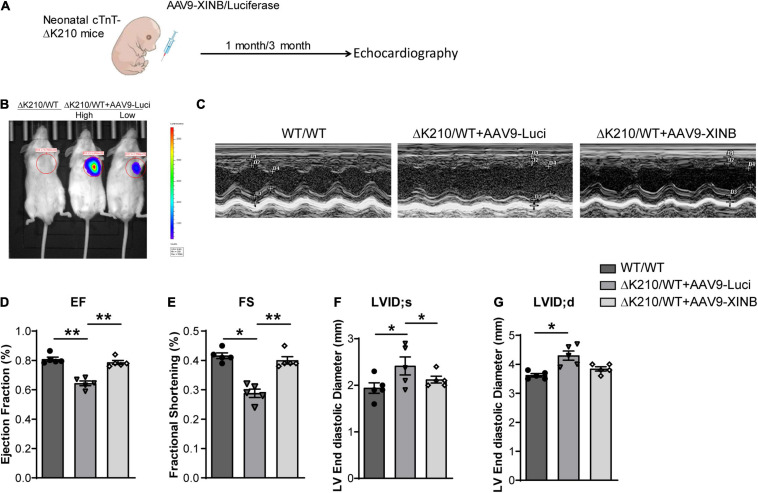
Cardiac-specific expression of XINB improved cardiac function of *TNNT2*-ΔK210 mice. **(A)** Schematic illustration of the design of XINB delivery through AAV9. **(B)** *In vivo* bioluminescence imaging of mice tested the efficiencies of AAV9-Luci with the cTnT promoter for cardiomyocyte-specific gene expression. 3-day-old neonatal mice were intraperitoneally injected with AAV9-Luci viruses (1 × 10^12^ viral genomes per mouse). Mouses without AAV injection were used as control. High: high dose. Low: low dose. **(C)** Representative M-mode echocardiography recordings of 3-months-old WT mice, AAV9-Luci injected *TNNT2*-ΔK210 mice, and AAV9-XINB injected *TNNT2*-ΔK210 mice. **(D)** Echocardiographic analysis of ejection fraction (EF), **(E)** fractional shortening (FS), **(F)** left ventricular end systolic internal diameter (LVID; s), and **(G)** left ventricular end diastolic internal diameter (LVID; d) of *TNNT2*-ΔK210 mice after XINB treatment (*n* = 5 per group). Data are represented as mean ± SEM. *P < 0.05, **P < 0.01.

To assess whether XINB could rescue the phenotype of DCM *in vivo*, we next used recombinant adeno-associated virus type 9 (AAV9) to specifically express XINB in the heart of neonatal *TNNT2*-ΔK210 DCM mice (Figure 5A). Plasmid AAV9: TNNT2: XINB (AAV9-XINB) with XINB driven by TNNT2 promoter was generated to express XINB specificially in cardiomyocytes, while AAV9: TNNT2: Luciferase (AAV9-Luci), in which XINB was replaced by luciferase, was used as control. To validate the efficiency of viral delivery, uninjected mice were used as controls. The luciferase protein was detected in hearts but not in any other organs of the AAV9-Luci treated mice, and also not found in the uninjected mice (Figure 5B). Functions of the wild type and *TNNT2*-ΔK210 transgenic mice were assessed using echocardiography at 1 and 3 months of age. The representative M-mode echocardiographic images at 3 months of age were shown in Figure 5C. LV ejection fraction (Figure 5D) and fractional shortening (Figure 5E) of the XINB-treated *TNNT2*-ΔK210 mice were significantly increased by 64.3 ± 3.7% to 78.6 ± 3.1% and 28.8 ± 3.1% to 40.0 ± 2.8% compared with control. The left ventricular (LV) end systolic and diastolic diameter of the mice was decreased by 2.5 ± 0.2 mm to 2.0 ± 0.06 mm and 4.3 ± 0.2 mm to 3.8 ± 0.1 mm at 3 months of age compared with that of the AAV9-luciferase injected *TNNT2*-ΔK210 transgenic mice (Figures 5F,G). These results suggested that the cardiac function of *TNNT2*-ΔK210 mice was significantly improved after XINB overexpression.

### XINB Improved Cardiac Pathological Phenotypes in the TNNT2-ΔK210 Mice

**FIGURE 6:**
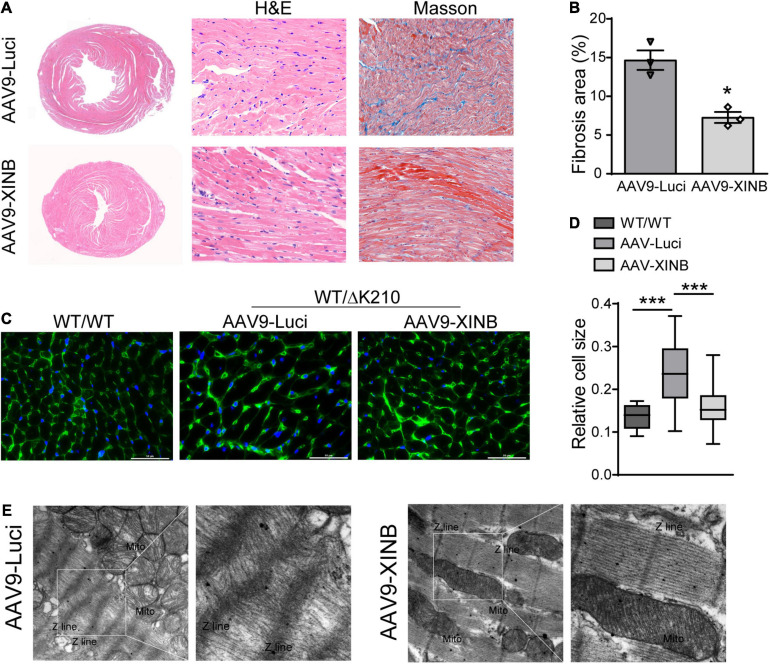
Morphological studies showed cardiac-specific expression of XINB in *TNNT2*-ΔK210 mice rescued DCM phenotypes. **(A)**, Representative H&E staining of the hearts from 3-months old control and AAV9-XINB injected *TNNT2*-ΔK210 mice. Masson’s trichrome staining showed less fibrosis in the mutant LV tissue when compared to control. **(B)** Quantification of interstitial fibrosis area in each group. **(C,D)** Wheat germ agglutinin (WGA; as an indicator of cardiomyocyte size) staining of left ventricle sections and quantification of myocyte sizes. Scale bars, 50 mm. **(E)** Ultrastructure of the left ventricular tissue sections from 3-months old mice revealed by transmission electron microscopy at ×6,000 magnification. AAV9-XINB treatment significantly improved the sarcomeric structure of *TNNT2*-ΔK210 mice. Data are represented as mean ± SEM. *P < 0.05, ***P < 0.001.

The morphological changes of heart ventricles and cardiomyocytes were further determined by histological examination (Figures 6A–E). Overexpression of XINB in the *TNNT2*-ΔK210 transgenic mice significantly reduced the ventricular dilatation and accumulation of collagen in the interstitial space compared with the typical histological changes observed in the heart tissues of the control *TNNT2*-ΔK210 mice (Figures 6A,B). At 3 months of age, *TNNT2*-ΔK210 mice also showed cardiomyocytes enlargement compared to WT mice. XINB overexpression prevented the development of heart dilation and hence recovered the size of myocytes (Figures 6C,D). Twisted Z-line, sparse myofilaments, and swollen mitochondria was also seen in the control *TNNT2*-ΔK210 mice hearts. However, the disrupted sarcomeric structures of heart was improved by XINB overexpression, which showed relative dense and aligned myofilaments with mitochondria arranged in the gap (Figure 6E). These changes indicated that the morphological phenotypes of DCM in *TNNT2*-ΔK210 mutant mice were partially rescued by cardiac-specific expression of XINB.

## Discussion

Detailed mechanisms for dilated cardiomyopathy caused by *TNNT2* gene mutation remain largely unknown. Current research using dilated myocardium from patients at a relative late disease stage might neglect the earliest molecular events which is key for DCM development. In this study, we used cardiomyocytes derived from hESCs which carried the DCM-causing *TNNT2*-ΔK210 mutation and confirmed XIN decreased compared with control. Furthermore, XINwas also decreased in DCM mouse heart. Overexpression of the repeat-containing isoform XINB in ΔK210 cardiomyocytes improved myofilaments organization and increased cell contractile force. Increase in the LVEF and LVFS after XINB treatment via AAV9 indicated that overexpression of XINB also improved heart functions *in vivo*. These results indicated XIN deficiency might be involved in the resulted loosely organized myofilament and systolic dysfunction caused by the *TNNT2*-ΔK210 mutation and overexpression of XINB could prevent DCM cardiac remodeling caused by the mutation.

Intra-exonic splicing of XIRP1 gene resulted in three XIN isoforms: XINA (a full length protein), XINB (lacks filamin C-binding carboxy terminus) and truncated XINC that lacks XIN repeats, β-catenin-binding region and most of functional regions (van der Ven et al., 2006). Because of the limitations of lentivirus or recombinant adeno-associated virus vector capacity, we used XINB in this study. And we speculate XINA, as a full length protein, will work as well as XINB. Previous study revealed XINC protein only existed in hypertrophic tissues by comparing normal and hypertrophic human cardiac samples (Otten et al., 2010), suggesting that this isoform might also be involved in the pathogenesis of cardiomyopathy. Organization of individual components into thick or thin filaments and their integration into M-bands and Z-lines are important for myofibrillar assembly (Claeys et al., 2009; Sanger et al., 2010). Consistent with our results, survey of microarray datasets in Gene Expression Omnibus (GEO) has revealed that *XIRP1* downregulated in failing hearts from human patients with diabetic or non-diabetic heart failure (GDS4314), and idiopathic DCM (GDS651) (Wang et al., 2014). XIN repeats as an actin-binding motif, directly bind actin filaments *in vitro* and cross link these filaments into networks in a similar manner as nebulin repeats. Within the XIN repeats, the overlapped β-catenin-binding domain, Ena/VASP binding domain DNA-binding domain and proline-rich region were identified from human XIN (van der Ven et al., 2006; Wang et al., 2012, 2013). XIN act as part of a complex that participate in the early morphogenesis of the heart and the maintenance of striated muscle integrity (Claeys et al., 2009). Deficiency of XIN might disrupt cytoskeletal protein arrangements and interactions, further weaken the stability of myofibril assembly and maintenance, thus leads to DCM.

In the present study, we were able to successfully rescue cardiac remodeling in a *TNNT2*-ΔK210 mouse model of dilated cardiomyopathy by overexpressing XIN. XIN deficiency might be a key mechanism of *TNNT2*-ΔK210 mutation-caused familial DCM and XIN could act as an interventional target for the relevant heart diseases.

## Data Availability Statement

The original contributions presented in the study are included in the article/Supplementary Material, further inquiries can be directed to the corresponding author/s.

## Ethics Statement

The animal study was reviewed and approved by Institutional Animal Care and Use Committee of the Fudan University.

## Author Contributions

BL, NS, QL, and CX conceived the research and wrote the manuscript. BL, YG, YZ, XZ, YL, and CZ performed the experiments and data analysis. All the authors contributed to the article and approved the submitted version.

## Conflict of Interest

The authors declare that the research was conducted in the absence of any commercial or financial relationships that could be construed as a potential conflict of interest.
