# Supplementary material for: Cardiac Overexpression of XIN Prevents Dilated Cardiomyopathy Caused by TNNT2 ΔK210 Mutation
Source: Front Cell Dev Biol. 2021 Jun 17;9:691749. doi: 10.3389/fcell.2021.691749 (PMC8247596; doi:10.3389/fcell.2021.691749)
Supplement: Supplementary file 1 [file Data_Sheet_1.docx]

**SUPPLEMENTARY MATERIAL**

**Cardiac Overexpression of XIN Prevents Dilated Cardiomyopathy Caused by *TNNT2* ΔK210 Mutation**

**Supplementary Table S1. Real-Time quantitative PCR Primers.**

| **Primer** | **Forward** | **Reverse** |
| --- | --- | --- |
| XIRP1-human | GTCAGTGCAACTCGCTGGAT | GGTGGGATAAGGTCTGGGGA |
| XINB-human | TCAGAGACAAGGTCCAAGTGG | CCTGTCCAATGCTGTCCAAGG |
| TNNT2-human | GGAGGAGTCCAAACCAAAGCC | TCAAAGTCCACTCTCTCTCCATC |
| ACTN2-human | CGTCGCTGACAGAGGTGC | CACCGATCATTGACATTCACAGC |
| MYH6-human | GCTGGTCACCAACAATCCCTA | CGTCAAAGGCACTATCGGTGG |
| MYH7-human | ACTGCCGAGACCGAGTATG | GCGATCCTTGAGGTTGTAGAGC |
| GAPDH-human | GGAGCGAGATCCCTCCAAAAT | GGCTGTTGTCATACTTCTCATGG |
| Xirp1-mouse | GGCCACCTCTCGAAAGTTTGA | CCTCATGGTTGTCTGTGTTGC |
| Gapdh-mouse | AGGTCGGTGTGAACGGATTTG | TGTAGACCATGTAGTTGAGGTCA |

**Supplementary Figures and Figure Legends**


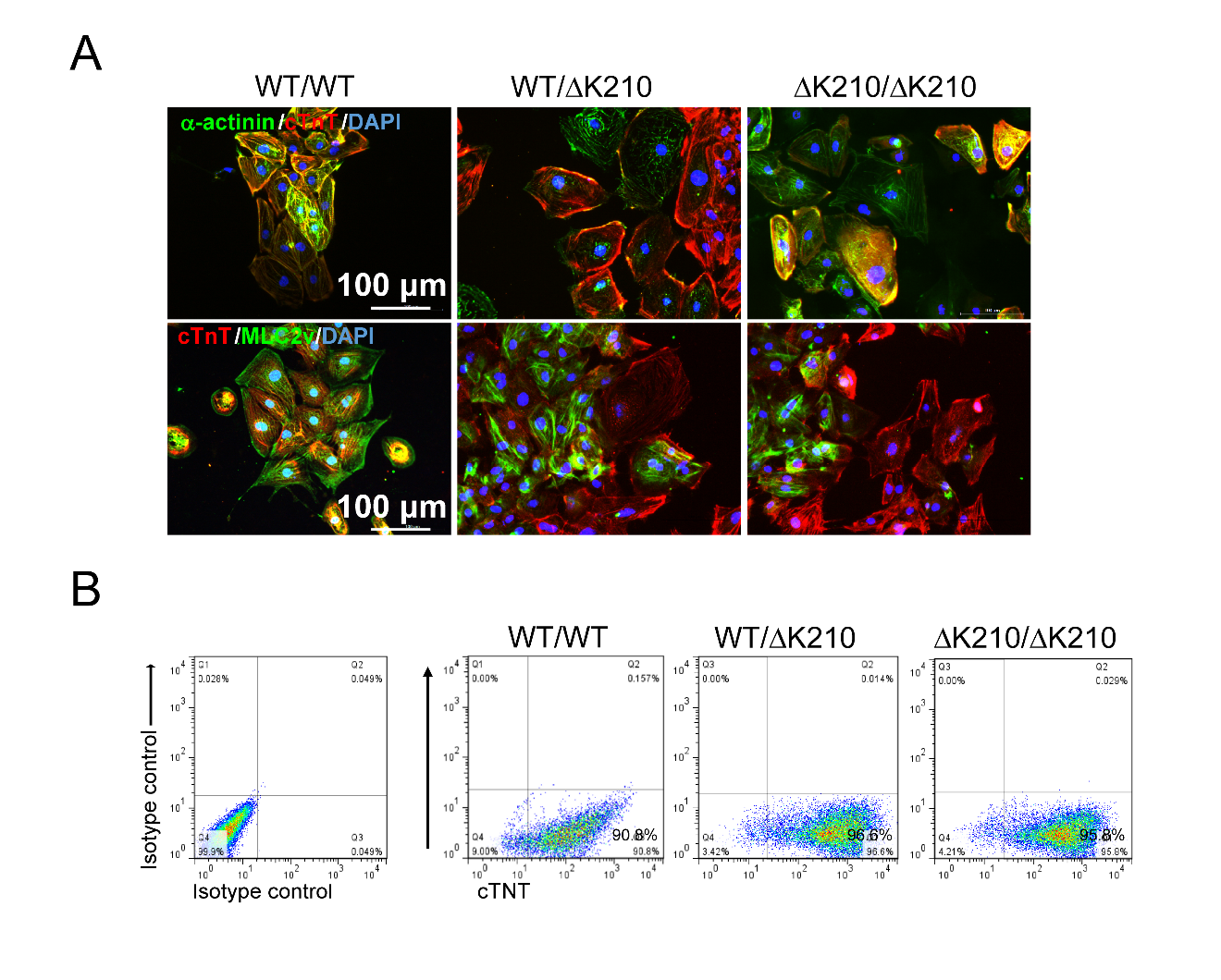


**Fig. S1, Establishment and characterization of WT and *TNNT2*-ΔK210 hESC-cardiomyocytes.** **A**. Immunostaining images showed WT and *TNNT2*-ΔK210 hESC-derived cardiomyocytes expressed cardiac-specific proteins cTnT, α-actinin and MLC2v. **B**. Flowcytometry analyses of cTnT+ cells in both unstained gating control and cTnT antibody-stained *TNNT2*-ΔK210 hESC cardiac differentiation showing similar efficiency (>90% differentiation efficiency) relative to the WT control. n≥3, 3 lines per group.


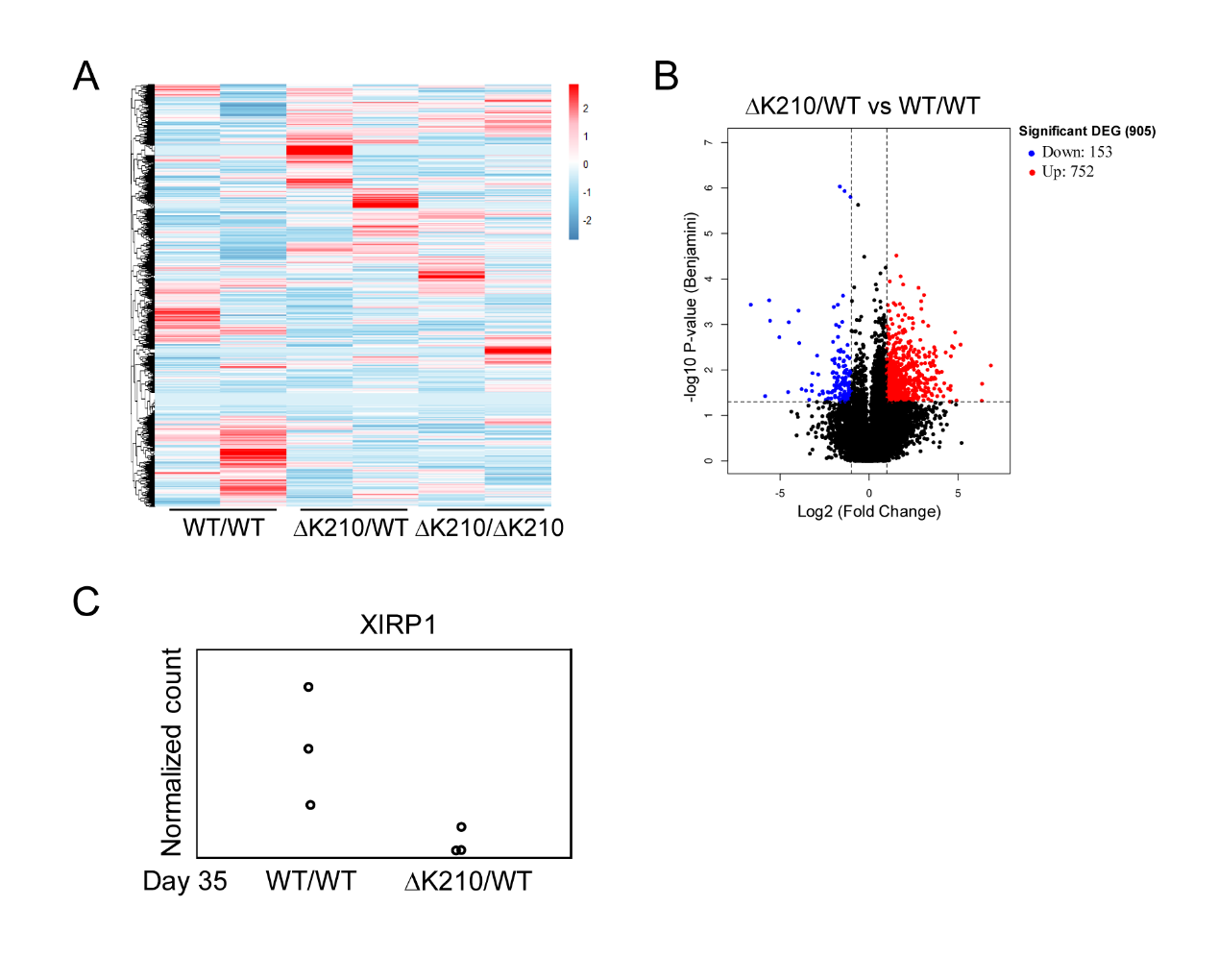


**Fig. S2, RNA-sequencing of WT/WT and ΔK210 cardiomyocytes**. **A**. Heat map of the whole transcriptomes of day35 WT/WT and ΔK210 cardiomyocytes. **B.** Volcano plot of differentially expressed genes between WT/WT and WT/ΔK210 cardiomyocytes. **C.** Differential expression analysis showed XIRP1 expression is down-regulated in WT/ΔK210 cardiomyocytes at day35 post differentiation based on the whole transcriptomic RNA-seq profiles.
